# Difficulties Experienced by Nurses in Psychiatric Hospitals During the Transfer of Psychiatric Inpatients With Physical Complications to General Hospitals: A Descriptive Cross‐Sectional Study

**DOI:** 10.1002/nop2.70509

**Published:** 2026-03-29

**Authors:** Rie Chiba, Misato Hirota, Yuta Hayashi, Maki Taniguchi, Yoshifumi Kido, Miyuki Notsu

**Affiliations:** ^1^ Human Health Sciences, Graduate School of Medicine Kyoto University Kyoto Japan; ^2^ Graduate School of Health Sciences Kobe University Kobe Japan; ^3^ Graduate School of Health Care Sciences Institute of Science Tokyo Tokyo Japan; ^4^ Department of Clinical Nursing Hamamatsu University School of Medicine Hamamatsu Japan; ^5^ Department of Nursing Arimakougen Hospital Kobe Japan

**Keywords:** communication, general hospitals, healthcare providers, mental disorders, patient transfer, physical complication, psychiatric hospitals, stigma

## Abstract

**Aim:**

To describe the difficulties experienced by nurses when transferring psychiatric inpatients with physical complications to general hospitals.

**Design:**

A cross‐sectional descriptive study.

**Methods:**

A total of 300 psychiatric hospitals were randomly selected from psychiatric hospitals nationwide in Japan. Nurses answered an anonymous, self‐administered questionnaire that quantitatively inquired about their experiences of difficulties when psychiatric inpatients with physical complications were transferred to general hospitals. The questionnaire included 17 items with five‐point options. Descriptive statistical analyses were performed.

**Results:**

A total of 242 responses from 80 hospitals were included in the analysis. More than 70% of the participants experienced somewhat or more difficulties because they felt that patients with mental illness were treated differently or hard to be understood and accepted when psychiatric inpatients with physical complications were transferred to general hospitals. Information sharing with general hospitals was related to significantly lower difficulties among nurses in psychiatric hospitals about the way healthcare providers in general hospitals viewed patients with mental illness, whereas the extent of cooperation and communication with general hospitals was not significantly associated with difficulties.

**Conclusion:**

Information sharing is related to lower difficulties in nurses in psychiatric hospitals when transferring inpatients to general hospitals.

**Reporting Methods:**

We followed STROBE statement in this study.

**Impact:**

Better linkage between psychiatric and physical healthcare, including improved referral pathways based on the information sharing, is important.

**Patient or Public Contribution:**

No patient or public contribution. However, three nurses in psychiatric hospitals engaged in clinical care were involved in the study because the survey was conducted with nurses at psychiatric hospitals.

## 1 Introduction

The number of patients with mental illness and their deaths has been increasing worldwide and will continue to increase in the future (Wu et al. 2023). Poor physical health among people with mental illness is a multifaceted, complicated and global issue. Although people with mental illness have an increased risk of various physical comorbidities (Launders et al. 2022; Momen et al. 2020; Na et al. 2024; Nielsen et al. 2021), psychiatric patients with physical complications do not necessarily receive appropriate physical screening, diagnosis or treatment, even when they require substantial physical treatment in a general medical department. This results in a much shorter life expectancy and personal, social and economic burdens across their lifespan (Ali et al. 2022; Correll et al. 2022; Momen et al. 2022). For example, patients with both mental illness and COVID‐19 infection showed higher mortality rates than those with only COVID‐19 (Wang et al. 2021).

## 2 Background

The high prevalence of common physical illnesses among psychiatric inpatients worldwide underscores the importance of adequate detection and medical treatment for physical comorbidities in inpatient settings (Goldman et al. 2020). However, physical treatment tends to be difficult, resulting in longer hospital stays or patient transfers (Han et al. 2022; Shields et al. 2020; Smith et al. 2021). In particular, Japan has a larger number and longer hospital stay of patients with mental illness than other Organization for Economic Cooperation and Development (OECD) countries, which exacerbates this issue (Okayama et al. 2020). In addition, inpatients in psychiatric care beds are aging dramatically, with approximately 60% of patients aged ≥ 65 years, resulting in a higher risk and prevalence of physical complications (Okayama et al. 2020). Furthermore, many private, single‐specialty psychiatric hospitals in Japan have made it difficult to manage cases across hospitals.

Previous studies have revealed that patient‐, provider‐ and system‐related factors affect physical care for psychiatric patients with physical comorbidities, ultimately influencing survival and quality of life (D'Alton et al. 2021; González‐Rodríguez et al. 2020; Kohn et al. 2022; Murphy et al. 2024). Patient‐related factors include higher rates of cognitive impairment, unawareness of physical problems, non‐adherence and the need for accompaniment. Provider‐related factors include stigma toward mental illness, lack of training and experience and the possibility of misinterpreting physical symptoms as psychosomatic complaints. System‐related factors include unavailability of equipment, understaffing, lack of patient advocacy and responsibility, lack of continuity of care and under‐resourcing of mental health care. Among these various factors with different aspects, stigma toward mental illness and patients with mental illness, combined with severe psychiatric symptoms, pose barriers and challenges for nurses in psychiatric hospitals, especially in acute ward settings, when inpatients with physical complications visit or transfer to general hospitals for seamless physical treatment (Brunero et al. 2020). Therefore, a collaborative relationship between nurses in psychiatric and general hospitals is crucial for improved physical care (Brunero et al. 2020; Mariano et al. 2021). For example, information sharing and cooperative and communicative relationships may be related to lower difficulties. However, to our knowledge, few studies have addressed our research questions, which are: (1) how do nurses in psychiatric hospitals experience difficulties when transferring psychiatric patients with physical comorbidities to general hospitals and (2) how do nurses' perceptions of difficulties vary by ward type and the extent of collaborative relationships with nurses in general hospitals? This would provide insights into how to seamlessly provide continuing care for psychiatric inpatients with comorbidities who require specialized physical treatment at general hospitals.

## 3 The Study

### 3.1 Aim

This study aimed to describe the difficulties experienced by nurses in psychiatric hospitals and how these difficulties vary by ward type and the extent of collaborative relationships with nurses in general hospitals when transferring psychiatric inpatients with physical complications to general hospitals.

## 4 Methods

### 4.1 Design, Sampling and Recruitment

A descriptive cross‐sectional survey was conducted. A total of 300 psychiatric hospitals were randomly selected from 1235 psychiatric hospitals nationwide, listed in the Ministry of Health, Labor and Welfare's database of health and medical institutions in Japan. Among all types of wards, the four main wards, that is, psychiatric emergency, psychiatric acute care, psychiatric general and psychiatric long‐term care wards, were selected as survey settings. While psychiatric emergency and psychiatric acute care wards are for acute patients, psychiatric general and psychiatric long‐term care wards are primarily for long‐stay patients. Owing to the past emphasis on institutional psychiatric care, Japan still has many psychiatric general and psychiatric long‐term care wards.

The participants were management nurses, that is, head nurses or deputy head nurses, who had experience actively transferring inpatients to a general hospital as responsible nurses. One management nurse per ward was asked to answer the questionnaire to avoid overrepresentation of nurses from a particular hospital or ward.

### 4.2 Data Collection

The survey was conducted in February and March 2022 by postal mail using anonymous, self‐administered questionnaires. The directors of nursing at each psychiatric hospital distributed the questionnaire to the head nurses or deputy head nurses in each ward. We asked participants to provide information about the characteristics of the ward to which they belonged: type of ward, number of patients admitted or transferred to the ward in the previous month, number of patients discharged from the ward or transferred to other hospitals in the previous month, average length of stay, percentage of voluntarily hospitalized patients, most common diagnoses and average age of inpatients.

We also asked nurses in psychiatric hospitals about their experiences of the difficulties when transferring inpatients with physical complications to general hospitals. Seventeen items were developed by the authors based on a literature review and discussions among six researchers in nursing and psychiatry, and three nurses at psychiatric hospitals, including a director of nursing. Considering the purpose of this study, the items comprised provider‐ and system‐related items. We conducted multiple discussions and refined the items, particularly based on the opinions of nurses at psychiatric hospitals. Before the survey, the authors, including the active nurses, checked and confirmed the face validity of each item.

Eleven provider‐related items comprised the attitudes and responses of healthcare providers in general hospitals (Cronbach's alpha = 0.87). Among them, four items (Items No. 1–4), such as ‘I had experienced difficulties because I felt that patients were treated differently because they were inpatients at psychiatric hospitals’, concerned how nurses in psychiatric hospitals feel difficulty in the way general hospitals view patients with mental illness. The remaining seven items (Items No. 5–11), such as ‘I had experienced difficulties when asked to ride an ambulance to transport a patient to a general hospital’, were related to how nurses in psychiatric hospitals are asked by healthcare providers in general hospitals to accompany patients with mental illness in various situations. On the other hand, six items were system‐related items (Cronbach's alpha = 0.75) involving two items regarding how nurses in psychiatric hospitals feel difficulty in human resources at psychiatric hospitals (Items No. 12, 13), such as ‘I had experienced difficulties because the hospital does not have nurses skilled in physical care’. Two items related to how nurses in psychiatric hospitals feel difficulty in the facilities or organization of psychiatric hospitals (Items No. 14, 15) included ‘I had experienced difficulties because the hospital does not have physical complications treatment ward’. And the other two items regarding how nurses in psychiatric hospitals feel difficulty in the locations of psychiatric hospitals (Items No. 16, 17) comprised ‘I had experienced difficulties because there are no general hospitals near our psychiatric hospital’. Each item was answered using a five‐point scale with 0 (none), 1 (a little), 2 (somewhat), 3 (quite) and 4 (very).

In addition, to assess the extent of information sharing and cooperation/communication with nearby general hospitals, we asked two items, ‘When a patient visits a hospital or is transferred to general hospitals, information about the patient is shared sufficiently between hospitals’ and ‘Cooperation/communication with nearby general hospitals are held regularly on a routine basis’. The former refers to the sharing of information about specific patients being transferred, while the latter refers to a network between hospitals in normal times. Respondents rated these two items on a five‐point scale: 1 (not applicable), 2 (not very applicable), 3 (neither), 4 (somewhat applicable) and 5 (very applicable).

### 4.3 Data Analysis

The characteristics of the ward and inpatients by ward type were presented as means, standard deviations, numbers and percentages. The difficulties experienced by nurses in psychiatric hospitals, as well as the extent of information sharing and cooperation/communication, were presented as numbers and percentages. To examine the differences in the extent of provider‐related difficulties related to attitudes and responses by healthcare providers at general hospitals (Items No. 1–11) by ward type, the extent of cooperation/communication and the extent of information sharing, the Kruskal–Wallis test and pairwise multiple comparisons were adopted. The scores of the extent of information sharing and cooperation/communication were transformed from a five‐point to a three‐point scale by converting ‘not applicable’ and ‘not very applicable’ to ‘No’, and ‘somewhat applicable’ and ‘very applicable’ to ‘Yes’ for three‐group comparison with an adequate sample size. Statistical significance was defined as a *p*‐value < 0.05 (two‐tailed). Statistical analyses were performed using the SPSS version 29 software (SPSS, Chicago, IL, USA).

### 4.4 Ethical Considerations

The participants read the purpose and methods of this study, including information that they would not be disadvantaged even if they refused to participate. This anonymous survey was conducted only with those who agreed to participate in the study after providing informed consent form. This study was approved by the Ethics Committee of Kobe University (No. 1064) and complied with the principles of the Declaration of Helsinki.

## 5 Results

### 5.1 Characteristics of the Wards and Inpatients

In this study, questionnaires were successfully sent to 299 of 300 randomly selected psychiatric hospitals, of which nurses from 80 hospitals nationwide responded and were included in the analysis (26.8%). We analysed the responses of 242 ward management nurses. Table 1 presents the characteristics of the wards and inpatients by ward type. The number of patients admitted (transferred) to the ward and the number of patients discharged from the ward or transferred to other hospitals per month were the highest in psychiatric emergency wards, followed by psychiatric acute care wards, psychiatric general wards and psychiatric long‐term care wards. Although the average length of stay in psychiatric emergency wards was 72.1 days, it was much longer in other types of wards (249.9–1568.1). The percentage of voluntarily hospitalized patients in psychiatric emergency wards was 28.3%, compared with approximately 50%–60% in other types of wards. Regardless of ward type, the most common diagnostic category for mental illness was F20–F29. The mean age of inpatients in psychiatric emergency wards was 52.4 years, whereas the mean age in other types of wards was in the 60s.

**TABLE 1:** The characteristics of the wards and inpatients by ward type (*N* = 232).

	Psychiatric emergency ward (*n* = 26), mean (SD)	Psychiatric acute care ward (*n* = 25), mean (SD)	Psychiatric general ward (*n* = 89), mean (SD)	Psychiatric long‐term care ward (*n* = 92), mean (SD)	Total (*N* = 232), mean (SD)
Number of beds in the ward	47.0 (13.6)	50.4 (7.9)	53.9 (11.8)	55.2 (6.9)	53.3 (10.2)
Number of patients admitted (transferred) to the ward per month	17.8 (13.6)	12.1 (8.7)	3.9 (6.1)	1.3 (1.6)	5.2 (8.4)
Number of patients discharged from the ward or transferred to other hospitals per month	14.4 (10.6)	8.9 (6.7)	3.0 (2.9)	1.9 (2.2)	4.4 (6.1)
Age of inpatients (years)	52.4 (7.3)	61.2 (9.4)	66.5 (9.8)	67.8 (8.5)	65.2 (10.2)
Length of stay (days)	72.1 (142.7)	249.9 (500.1)	876.6 (1195.3)	1568.1 (1567.9)	978.0 (1370.2)
% of voluntarily hospitalized patients	28.3%	61.2%	49.4%	60.3%	50.5%
Most common diagnostic category (ICD‐10)	F20–F29	F20–F29	F20–F29	F20–F29	F20–F29

*Note:* Ten respondents were excluded from the analysis due to missing response on their wards.

### 5.2 The Difficulties Among Nurses in Psychiatric Hospitals When Transferring Inpatients to General Hospitals

Table 2 shows the extent of the difficulties experienced by psychiatric nurses in the past year when psychiatric inpatients with physical complications were transferred to general hospitals. Among the provider‐related items on attitudes and responses by healthcare providers at general hospitals (Items No. 1–11), more than 70% of participants experienced difficulties of somewhat or more because they felt that patients with mental illness were treated differently or were hard to understand and accept (Items No. 1, 2). In addition, 57.8% of the respondents had difficulties of somewhat or more in getting nurses at general hospitals to understand patients' mental states (Item 3). Furthermore, 47.0% of the respondents experienced somewhat or more difficulties in transferring non‐voluntary hospitalized patients to general hospitals (Item 4). More than 20% of respondents experienced somewhat or more difficulties due to the demands for direct assistance, such as ambulance escorts, attending patients in general hospitals or even consenting to treatment on behalf of patients (Items No. 5–11), except for waiting until a patient's surgery started with 12.0% (Item No. 10). The results also showed that difficulties of somewhat or more related to insufficient human resources or issues in the facilities or organization of psychiatric hospitals were observed in more than 40% of the respondents (Items No. 12–15), whereas difficulties of somewhat or more related to issues in the locations of psychiatric hospitals were experienced in 15.3%–21.1% of the respondents (Items No. 16, 17).

**TABLE 2:** The difficulties of psychiatric nurses in the past year when transferring psychiatric inpatients with physical complications to general hospitals (*N* = 242).

		No, *n* (%)	A little, *n* (%)	Somewhat, *n* (%)	Quite, *n* (%)	Very, *n* (%)	Unknown, *n* (%)
**Provider‐related items (Cronbach's alpha = 0.87)**							
(1) How nurses in psychiatric hospitals feel difficult about the way nurses in general hospitals look at patients with mental illness							
1	I had experienced difficulties because I felt that patients were treated differently because they were inpatients at psychiatric hospitals.	37 (15.3)	**56 (23.1)**	**59 (24.4)**	**60 (24.8)**	30 (12.4)	0
2	I had experienced difficulties because I felt that general hospitals were reluctant to accept patients because they were inpatients at psychiatric hospitals.	19 (7.9)	**53 (21.9)**	**52 (21.5)**	**76 (31.4)**	42 (17.4)	0
3	I had experienced difficulties getting staff to understand the mental state of patients being transferred to general hospitals.	41 (16.9)	**61 (25.2)**	**62 (25.6)**	**56 (23.1)**	22 (9.1)	0
4	I had experienced difficulties with barriers to transferring non‐voluntary hospitalized patients to physical hospitals.	**80 (33.1)**	42 (17.4)	40 (16.5)	48 (19.8)	26 (10.7)	6 (2.5)
(2) How nurses in psychiatric hospitals are asked by nurses in general hospitals to accompany patients with mental illness in various situations							
5	I had experienced difficulties when asked to ride an ambulance to transport a patient to a general hospital.	**149 (61.6)**	35 (14.5)	23 (9.5)	22 (9.1)	11 (4.5)	2 (0.8)
6	I had experienced difficulties when asked to accompany patients or act as a mediator when they were transferred to general hospitals.	**115 (47.5)**	**55 (22.7)**	43 (17.8)	19 (7.9)	10 (4.1)	0
7	I had experienced difficulties when asked to be present while the patient's consent form was being filled out.	**145 (59.9)**	35 (14.5)	27 (11.2)	21 (8.7)	13 (5.4)	1 (0.4)
8	I had experienced difficulties when asked to assist, be present, or accompany patients during an examination at general hospitals.	**118 (48.8)**	47 (19.4)	41 (16.9)	20 (8.3)	15 (6.2)	1 (0.4)
9	I had experienced difficulties when asked to consent to a patient's treatment such as surgery.	**149 (61.6)**	23 (9.5)	23 (9.5)	18 (7.4)	27 (11.2)	2 (0.8)
10	I had experienced difficulties when asked to wait until a patient's surgery was about to start.	**196 (81.0)**	13 (5.4)	10 (4.1)	13 (5.4)	6 (2.5)	4 (1.7)
11	I had experienced difficulties when asked to confirm a patient's will for life‐prolonging treatment.	**170 (70.2)**	16 (6.6)	24 (9.9)	12 (5.0)	16 (6.6)	4 (1.7)
**System‐related items (Cronbach's alpha = 0.75)**							
(1) How nurses in psychiatric hospitals feel difficulties in human resources at psychiatric hospitals							
12	I had experienced difficulties because the hospital does not have nurses skilled in physical care.	**77 (31.8)**	**60 (24.8)**	**77 (31.8)**	26 (10.7)	2 (0.8)	0
13	I had experienced difficulties because the hospital does not have a doctor who specialized in physical medicine.	**82 (33.9)**	43 (17.8)	44 (18.2)	**50 (20.7)**	2 (9.5)	0
(2) How nurses in psychiatric hospitals difficulties in the facilities or organization of psychiatric hospitals							
14	I had experienced difficulties because the hospital does not have physical complications treatment ward.	**91 (37.6)**	42 (17.4)	32 (13.2)	**52 (21.5)**	23 (9.5)	2 (0.8)
15	I had experienced difficulties because the hospital does not have adequate medical equipment such as CT scans.	**84 (34.7)**	**52 (21.5)**	**54 (22.3)**	33 (13.6)	19 (7.9)	0
(3) How nurses in psychiatric hospitals difficulties in the locations of psychiatric hospitals							
16	I had experienced difficulties because there are no general hospitals near our psychiatric hospital.	**183 (75.6)**	22 (9.1)	25 (10.3)	7 (2.9)	5 (2.1)	0
17	I had experienced difficulties with poor transportation access near our psychiatric hospital.	**155 (64.0)**	36 (14.9)	34 (14.0)	13 (5.4)	4 (1.7)	0

*Note:* Bold figures indicate ≥ 20%.

### 5.3 The Extent of Information Sharing and Cooperation/Communication

The items regarding the extent of information sharing about patients with nearby general hospitals provided the following distribution: not applicable (*n* = 11), not very applicable (*n* = 36), not either (*n* = 84), somewhat applicable (*n* = 90), very applicable (*n* = 18) and no answer (*n* = 3). Another item regarding cooperation and communication with nearby general hospitals provided the following distribution: not applicable (*n* = 27), not very applicable (*n* = 50), not either (*n* = 78), somewhat applicable (*n* = 63), very applicable (*n* = 21) and no answer (*n* = 3).

### 5.4 The Differences of Difficulties Among Nurses in Psychiatric Hospitals When Transferring Inpatients to General Hospitals by Conditions

The extent of provider‐related difficulties during transfer did not differ by ward type, except for Item No. 9 (Table 3). Only on the item regarding difficulties when asked to consent to a patient's treatment, such as surgery, the mean score in the psychiatric emergency ward was significantly lower than that in the other three wards. On the other hand, information sharing with general hospitals was related to significantly lower difficulties among nurses in psychiatric hospitals about the way healthcare providers in general hospitals view patients with mental illness (Table 4). In contrast, the extent of cooperation and communication with general hospitals was not significantly associated with difficulties among nurses in psychiatric hospitals (Table 5).

**TABLE 3:** The difficulties of psychiatric nurses in the past year when transferring psychiatric inpatients with physical complications to general hospitals by ward type (*N* = 232).

		Psychiatric emergency ward (*n* = 26), mean (SD)	Psychiatric acute care ward (*n* = 25), mean (SD)	Psychiatric general ward (*n* = 89), mean (SD)	Psychiatric long‐term care ward (*n* = 92), mean (SD)	*p*
1	I had experienced difficulties because I felt that patients were treated differently because they were inpatients at psychiatric hospital.	1.9 (1.2)	2.4 (1.2)	1.9 (1.3)	2.0 (1.3)	0.282
2	I had experienced difficulties because I felt that general hospitals were reluctant to accept patients because they were inpatients at psychiatric hospitals.	2.4 (1.0)	2.7 (1.2)	2.3 (1.1)	2.2 (1.3)	0.314
3	I had experienced difficulties getting staff to understand the mental state of patients being transferred to general hospitals.	1.9 (1.2)	2.1 (1.3)	1.9 (1.2)	1.8 (1.2)	0.717
4	I had experienced difficulties with barriers to transferring non‐voluntary hospitalized patients to physical hospitals.	1.8 (1.2)	1.7 (1.6)	1.6 (1.4)	1.5 (1.4)	0.526
5	I had experienced difficulties when asked to ride an ambulance to transport a patient to a general hospital.	0.9 (1.0)	0.9 (1.1)	0.8 (1.2)	0.7 (1.3)	0.468
6	I had experienced difficulties when asked to accompany patients or act as a mediator when they were transferred to general hospitals.	1.0 (1.0)	1.2 (1.2)	1.0 (1.2)	1.0 (1.2)	0.755
7	I had experienced difficulties when asked to be present while the patient's consent form was being filled out.	0.5 (0.9)	0.8 (1.1)	1.0 (1.4)	0.9 (1.3)	0.478
8	I had experienced difficulties when asked to assist, be present or accompany patients during an examination at general hospitals.	1.1 (1.1)	1.2 (1.3)	1.1 (1.3)	1.0 (1.3)	0.805
9	I had experienced difficulties when asked to consent to a patient's treatment such as surgery.	0.3 (0.7)	0.7 (1.4)	1.2 (1.5)	1.1 (1.5)	**0.033** *
10	I had experienced difficulties when asked to wait until a patient's surgery was about to start.	1.2 (0.5)	0.6 (1.3)	0.4 (1.1)	0.5 (1.1)	0.545
11	I had experienced difficulties when asked to confirm a patient's will for life‐prolonging treatment.	0.3 (0.7)	0.8 (1.4)	0.7 (1.2)	0.8 (1.4)	0.525

*Note:* Ten respondents were excluded from the analysis due to a missing response on their wards. Bold values indicate Significant *p* values.

* The mean score in Psychiatric Emergency Ward was significantly lower than that in the other three wards on Item 15.

**TABLE 4:** The difficulties of psychiatric nurses in the past year when transferring psychiatric inpatients with physical complications to general hospitals by whether information sharing with general hospitals was held or not (*N* = 239).

		Information sharing with general hospitals			*p*
		No (*n* = 47; 19.7%), mean (SD)	Neither (*n* = 84; 35.1%), mean (SD)	Yes (*n* = 108; 45.2%), mean (SD)	
1	I had experienced difficulties because I felt that patients were treated differently because they were inpatients at psychiatric hospital.	2.4 (1.2)	2.3 (1.2)	1.5 (1.1)	**< 0.001** Yes < No; Yes < Neither
2	I had experienced difficulties because I felt that general hospitals were reluctant to accept patients because they were inpatients at psychiatric hospitals.	2.7 (1.0)	2.6 (1.2)	1.9 (1.2)	**< 0.001** Yes < No; Yes < Neither
3	I had experienced difficulties getting staff to understand the mental state of patients being transferred to general hospitals.	2.3 (1.2)	2.1 (1.3)	1.4 (1.1)	**< 0.001** Yes < No; Yes < Neither
4	I had experienced difficulties with barriers to transferring non‐voluntary hospitalized patients to physical hospitals.	2.0 (1.5)	1.7 (1.5)	1.2 (1.3)	**0.003** Yes < No; Yes < Neither
5	I had experienced difficulties when asked to ride an ambulance to transport a patient to a general hospital.	1.0 (1.3)	1.0 (1.3)	0.5 (1.0)	**0.005** Yes < Neither
6	I had experienced difficulties when asked to accompany patients or act as a mediator when they were transferred to general hospitals.	1.2 (1.2)	1.1 (1.2)	0.8 (1.0)	**0.020** Yes < No; Yes < Neither
7	I had experienced difficulties when asked to be present while the patient's consent form was being filled out.	0.9 (1.2)	1.0 (1.4)	0.6 (1.1)	0.105
8	I had experienced difficulties when asked to assist, be present or accompany patients during an examination at general hospitals.	1.1 (1.2)	1.3 (1.4)	0.8 (1.1)	0.091
9	I had experienced difficulties when asked to consent to a patient's treatment such as surgery.	1.0 (1.4)	1.0 (1.4)	0.9 (1.4)	0.562
10	I had experienced difficulties when asked to wait until a patient's surgery was about to start.	0.4 (0.9)	0.6 (0.7)	0.3 (0.7)	0.239
11	I had experienced difficulties when asked to confirm a patient's will for life‐prolonging treatment.	0.9 (1.3)	0.7 (1.3)	0.6 (1.2)	0.294

*Note:* Three respondents were excluded from the analysis due to a missing response on information sharing. Bold values indicate Significant *p* values.

**TABLE 5:** The difficulties of psychiatric nurses in the past year when transferring psychiatric inpatients with physical complications to general hospitals by whether cooperation and communication with nearby general hospitals were held or not (*N* = 239).

		Cooperation and communication with nearby general hospitals			*p*
		No (*n* = 77; 32.2%), mean (SD)	Neither (*n* = 78; 32.6%), mean (SD)	Yes (*n* = 84; 35.2%), mean (SD)	
1	I had experienced difficulties because I felt that patients were treated differently because they were inpatients at psychiatric hospital.	2.1 (1.2)	1.9 (1.3)	1.9 (1.3)	0.588
2	I had experienced difficulties because I felt that general hospitals were reluctant to accept patients because they were inpatients at psychiatric hospitals.	2.4 (1.1)	2.3 (1.2)	2.2 (1.3)	0.591
3	I had experienced difficulties getting staff to understand the mental state of patients being transferred to general hospitals.	2.0 (1.2)	1.8 (1.2)	1.7 (1.2)	0.483
4	I had experienced difficulties with barriers to transferring non‐voluntary hospitalized patients to physical hospitals.	1.6 (1.4)	1.5 (1.4)	1.6 (1.4)	0.967
5	I had experienced difficulties when asked to ride an ambulance to transport a patient to a general hospital.	0.7 (1.1)	0.9 (1.3)	0.8 (1.2)	0.755
6	I had experienced difficulties when asked to accompany patients or act as a mediator when they were transferred to general hospitals.	0.9 (1.2)	1.1 (1.2)	0.9 (1.1)	0.590
7	I had experienced difficulties when asked to be present while the patient's consent form was being filled out.	0.7 (1.1)	0.9 (1.3)	0.9 (1.2)	0.507
8	I had experienced difficulties when asked to assist, be present or accompany patients during an examination at general hospitals.	0.8 (1.1)	1.1 (1.3)	1.1 (1.2)	0.463
9	I had experienced difficulties when asked to consent to a patient's treatment such as surgery.	0.8 (1.3)	0.9 (1.4)	1.2 (1.5)	0.462
10	I had experienced difficulties when asked to wait until a patient's surgery was about to start.	0.4 (1.0)	0.5 (1.1)	0.3 (0.9)	0.557
11	I had experienced difficulties when asked to confirm a patient's will for life‐prolonging treatment.	0.7 (1.2)	0.7 (1.2)	0.7 (1.3)	0.943

*Note:* Three respondents were excluded from the analysis due to a missing response on cooperation and communication.

## 6 Discussion

This cross‐sectional survey revealed that a high proportion of nurses from all ward types in psychiatric hospitals experienced various difficulties when inpatients with physical complications were transferred to general hospitals. Among these, difficulties related to the attitudes and responses of healthcare providers in general hospitals were commonly observed. Information sharing with general hospitals was associated with fewer difficulties related to the way general hospitals viewed patients with mental illness and the request by general hospitals to accompany patients with mental illness; however, the extent of cooperation and communication with general hospitals was not significantly associated with difficulties.

More than 70% of the nurses had difficult experiences in which they felt that psychiatric inpatients were treated differently or were reluctant to be accepted. This result is consistent with the findings of a previous qualitative study that revealed that healthcare providers at general hospitals tend to feel that the transfer of psychiatric patients with physical comorbidities from psychiatric hospitals to general hospitals is not reasonable (Kohn et al. 2022). Careful communication with patients and care for mental health symptoms, which are important in psychiatric and mental health nursing, are not necessarily the main focus of care for patients with physical illnesses in general hospitals. Therefore, difficulties in responding to active psychiatric symptoms in general hospitals increase the burden and anxiety of nurses and decrease their confidence (Ngune et al. 2024). Given that previous studies have also shown that healthcare providers are just as susceptible to stigmatizing beliefs and behaviours toward people with mental illness (Kohn et al. 2022; Leahy et al. 2021), it is necessary to provide educational support for nurses in general hospitals to increase their knowledge to make the required assessments and provide optimal mental health care (Johanna et al. 2022; Mudgal et al. 2020).

Moreover, more than 20% of respondents were asked to accompany patients or confirm their will on their behalf. One can argue that such difficulties among nurses in psychiatric hospitals may occur when they are expected to or end up taking on roles outside of their original duties. In addition to training, clarifying and adjusting the scope of each role based on cooperative relationships should be considered.

Contrary to our expectations, the extent of difficulties related to attitudes and responses by healthcare providers at general hospitals during transfers did not differ by ward type. The more severe the psychiatric symptoms, the more difficult it should be to transfer a patient to a general hospital. Moreover, acute care wards tend to experience a shortage of nursing staff (Frawley and Culhane 2024). However, the findings of this study indicate that almost the same level of difficulty can occur, regardless of the severity of mental illness or possible understaffing at psychiatric hospitals. It can be argued that not only negative attitudes toward substantial psychiatric symptoms at general hospitals and fewer human resources at psychiatric hospitals, but also other system‐related factors at general hospitals, such as the treatment environment, understaffing and under‐resourcing of mental health care, may also have an impact. Only one item that showed a significantly lower score in the psychiatric emergency ward was related to consent to a patient's treatment, such as surgery. As it was the only ward in which the average age was in their 50s, there might have been fewer elderly inpatients with physical complications who required surgery.

Information sharing about inpatients between psychiatric and general hospitals was related to significantly fewer difficulties experienced by nurses in psychiatric hospitals. Shields et al. (2020) suggested that it is critically important for healthcare providers to exchange health information when psychiatric patients with physical comorbidities are transferred from inpatient psychiatric units to another to ensure that they receive adequate and informed follow‐up care. Although the importance of information sharing was consistent with earlier studies, this study also showed that only 44.6% of the respondents answered that they were involved in it. Therefore, designing a system that can integrate psychiatric and physical healthcare, including improved referral pathways based on the information sharing, may be a future topic (Björk Brämberg et al. 2018; Holland and Hudson‐Jessop 2023).

Interestingly, this study showed that the extent of cooperation and communication with general hospitals in normal times was not significantly associated with difficulties among nurses in psychiatric hospitals. Even if sufficient communication is maintained between hospitals, it may not contribute to reducing the difficulties. Rather than simply increasing opportunities for communication, innovative approaches, including patients' perspectives on fostering collaboration and networking across different sectors, disciplines and hospitals, may be more important for the integration of timely mental health and physical care (Leahy et al. 2021, 2024). Previous studies have also reported the significance of collaboration between nurses and other professionals, such as doctors (Saint‐Pierre et al. 2018). While this study focused on information sharing and interactions between nurses at different hospitals, collaboration between multiple professions at different hospitals may also affect the difficulties faced by nurses in psychiatric hospitals, which may be an issue for future research.

### 6.1 Strength and Limitations of the Work

One of the strengths of this study is that we used random sampling to extract the psychiatric hospitals in which we conducted the survey, thus preventing possible bias in the settings included. Second, three nurses in psychiatric hospitals working in clinical care were involved in the study. Their active engagement greatly contributed to the development of items to comprehensively evaluate the experienced difficulties.

This study has several limitations. First, the demographic and occupational characteristics of the respondents were not investigated. Second, nurses from 80 of the 300 randomly selected psychiatric hospitals responded to the questionnaire. Therefore, the results may not fully reflect the difficulties experienced by nurses in psychiatric hospitals under various circumstances. In addition, because nurses in psychiatric general wards and psychiatric long‐term care wards accounted for approximately 75% of the total participants analysed, it is likely that the results strongly reflected the characteristics of the difficulties experienced by nurses in these wards, resulting in weakened generalizability of the results. Finally, although the questionnaire items showed high internal consistency, validation of psychometric properties, including factor analysis, was not conducted in this study.

### 6.2 Recommendations for Further Research

Future studies using validated scales and larger sample sizes are needed to rigorously verify the various difficulties and related factors. In particular, examining the factors, such as nurses' clinical experience, educational background and the regional location of the hospital, that are related to the sense of difficulty during the transfer of inpatients should be the focus of future studies. In addition, including the perspectives of nurses in general hospitals and patients is important to explore better care for psychiatric patients with physical comorbidities.

## 7 Conclusion

More than 70% of the nurses at psychiatric hospitals experienced various difficulties when inpatients with physical complications were transferred to general hospitals. Among these, difficulties related to the responses of healthcare providers at general hospitals were commonly observed. Information sharing would be beneficial in reducing difficulties between psychiatric and general hospitals.

## Author Contributions

Study design: R.C., M.H., Y.H., M.T., Y.K. and M.N. Data collection: R.C., M.H. and Y.H. Data analysis: R.C. Manuscript writing: R.C. Proof reading: R.C., M.H., Y.H., M.T., Y.K. and M.N.

## Funding

This study was supported by Research Grant at Arimakougen Psychiatry Research Center (2021).

## Conflicts of Interest

The authors declare no conflicts of interest.

## Data Availability

The data that support the findings of this study are available from the corresponding author upon reasonable request.
